# Quantitative logics for directed evolution of an asexual population

**DOI:** 10.1371/journal.pone.0354488

**Published:** 2026-07-29

**Authors:** Kangbien Park

**Affiliations:** Independent Researcher, Seoul, Republic of Korea; Qingdao University, CHINA

## Abstract

Directed evolution of asexual populations is expected to offer a wide range of benefits to humanity. Achieving efficient directed evolution (DE) requires a quantitative formulation of experimental methodologies—or logics—that can address potential challenges throughout the evolutionary process. In this article, ten logics designed for resolving such difficulties when performing DE are introduced. To illustrate their application, a hypothetical scenario is considered in which an imaginary asexual population limb evolves into a wing, using a matrix-based discretization method to represent the trait of interest. Specifically, based on the operator model, fifty simulation iterations with logics applied and thirty iterations without logics were done. The results indicate that the introduced logics can accelerate the evolutionary process by roughly 2.6 times, while achieving an average accuracy for reaching the objective trait of approximately 82%. Based on these findings, key considerations for implementing quantitative logic-based DE are discussed, along with approaches for improving alignment between the final outcome and the target reference trait. Moreover, factors that could contribute to making the quantitative logic-based DE process more practical and rigorous are also examined.

## 1 Introduction

Directed evolution (DE) of asexual populations is an active area of research and is expected to bring diverse benefits to humanity [1,2]. Despite substantial experimental progress in implementing DE through biological and chemical techniques [3,4], quantitative modeling remains a crucial component of the DE process [5–7]. In particular, mathematical formulations enable rigorous analysis to overcome key challenges associated with evolutionary experiments. For example, when a population becomes trapped at an undesired local optimum on the fitness landscape, intervention methods are required to steer the population along pathways that descend the landscape. Moreover, if the evolutionary process takes too long to reach the target trait, means to accelerate the trajectory become necessary. Additionally, mathematical formulations provide insight into how changes in environmental conditions or differences in evolutionary speed influence the overall success probability of the evolutionary process, as these outcomes are ultimately governed by stochastic evolutionary dynamics. To this end, predictive tools that estimate the evolutionary timescale based on mathematically defined experimental designs, and that suggest shortcuts informed by such predictions, are essential. Rigorously achieving this requires precise mathematical modeling of both the trait and its associated potential. Fortunately, an algebraic representation for modeling the evolution of phenotypical traits in asexual populations has recently been proposed [8]. Specifically, if one defines an algebra (i.e., an algebraic set together with a well-defined operation that closes the set) that effectively represents the phenotypic trait of interest, the model can reliably predict the asexual evolutionary dynamics of that trait. As the model is based on the traveling wave model [9], which has been extensively studied both experimentally and theoretically, the predictions derived from the model are highly reliable and can be effectively applied to explain realistic biological evolution.

In this context, with the growing body of knowledge in evolutionary dynamics and population genetics, along with the rapid increase in computational power driven by advances in computer science and AI, it has become increasingly promising to apply quantitative methodologies to the DE of asexual populations [10–12]. Therefore, numerical expression that corresponds to the artificial intervention experimental methodologies to direct the evolution, or *logics*, can serve as a foundation for systematically guiding and optimizing the DE process.

In this article, ten plausible logics for artificially directing evolution are mathematically formulated, and the corresponding timescale for DE process are estimated. To accomplish this, an algebraic model based on an $n_{1}\times n_{2}\times n_{3}$ matrix is used to represent the trait of interest, capturing the 3 dimensional morphological structure of an imaginary asexual population limb in the *x*-, *y*-, and *z*-directions. This representation is constructed using a discretization method that converts a continuous geometric structure into a matrix-based form. The results show that these logics can accelerate the evolutionary process by an order of magnitude. In addition, the potential implementation of these logics in laboratory settings is discussed.

## 2 Theory and methodology

For the overall analysis, an algebraic model [8] based on the operator framework is employed [13]. Here, the algebraic model assumes a situation in which mutations of interest occur only within the algebraic set, while the potential is expressed with the algebra elements. Specifically, the potential is defined as $\Phi =F_{\max}-F$, where *F* denotes the fitness landscape [8].

**Fig 1:**
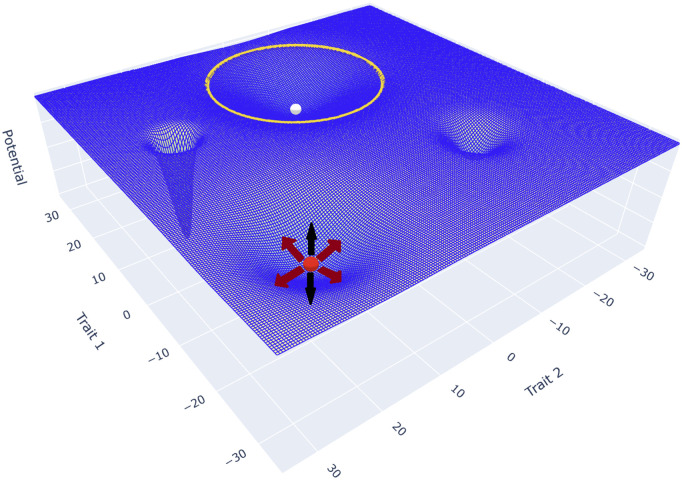
Illustration of the objective of directed evolution. In this two-dimensional vector evolution example, the white ball represents the target vector. The goal of the directed evolution process is to guide the red ball (the current state) to the position of the white ball by moving it along the potential surface.

Fig 1 illustrates the scheme for directing the evolution of a two-dimensional vector using the algebraic model to represent the evolution of the corresponding trait. Here, the red arrows indicate possible mutation directions within the plane, while the black arrows represent mutations in the *z*-direction (or any other higher-dimensional direction), which are forbidden under the assumption of a closed algebraic set. In this setting, the goal of DE is to guide the current population state (red ball) toward the target state (white ball). For more specific information regarding such algebraic evolution, refer to [8].


$$\begin{array}{c} P_{establishment\;per\;generation}\sim -\Delta \Phi \cdot m\cdot N(t), \end{array}$$ (1)

However, because the potential is convex near the red ball, *a quantitative formulation of experimental methodologies to intervene in the DE process*—or “*logics*”—are required to push the ball upward along the potential Φ. Otherwise, an establishment event leading to a higher potential level will not occur naturally, since equation (1), which describes the probability of establishment [8], implies that $P_{establishment\;per\;generation}<0$ (i.e., impossible) whenever ΔΦ>0. That is, the population always evolves in a direction in which the potential decreases, just as a ball rolls down a slope. Furthermore, even after the population reaches the flat region, additional guidance is required for the population to reach the yellow ring. Once the population arrives at the ring, the evolutionary process naturally converges to the target position, as described by equation (2) [8], where $A_{i}$ represents the algebra of interest in the *i*-th generation, $m_{A_{i}\leftarrow A_{i-1}}$ is the mutation rate from algebra $A_{i-1}$ to $A_{i}$, and $\Delta \Phi_{A_{i}\leftarrow A_{i-1}}$ is the potential difference between $A_{i-1}$ and $A_{i}$.


$$\begin{array}{c} T_{establishment}=\left\{ \begin{array}{c} T_{{est}_{A_{i+1}}}=\frac{1}{\left|\Delta \Phi_{A_{i}\leftarrow A_{i-1}}\right|}\cdot \ln\;\left(\frac{(\Delta \Phi_{A_{i}\leftarrow A_{i-1}}{)}^{2}}{k\cdot m_{A_{i}\leftarrow A_{i-1}}\cdot |\Delta \Phi_{A_{i+1}\leftarrow A_{i}}|}\right),\;i\geq 1,\\ T_{{est}_{A_{1}}}=\frac{1}{N\cdot m_{A_{1}\leftarrow A_{0}}\cdot |\Delta \Phi_{A_{1}\leftarrow A_{0}}|}. \end{array}\right. \end{array}$$ (2)

To achieve this, ten logics are introduced in this article: the logic of switching traits, the logic of towing from linkage, the logic of towing from phenotypic dependence, the logic of towing from pleiotropy, the logic of emergence, the logic of varying mutation rate, the logic of paste, the logic of positive potential, the logic of negative potential, and the logic of explicit modification. While other logics may also be devised to manipulate evolutionary trajectories, this article focuses on these ten.

### 2.1 First logic: switching traits

**Fig 2:**
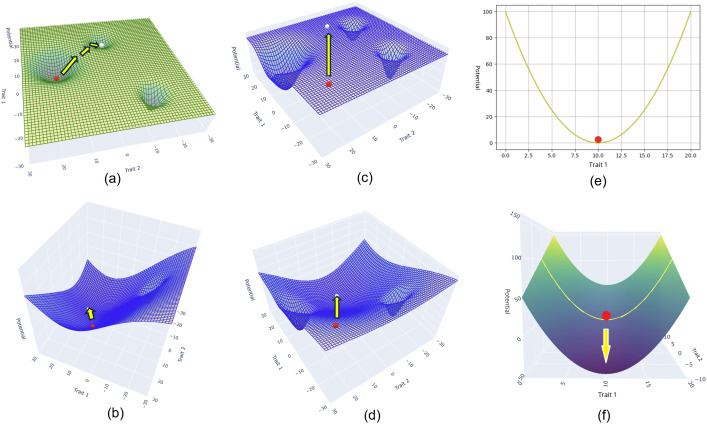
Pictorial representation of the logics in the 2D vector case. Panels (a) and (b) represent the logic of switching traits as well as the three variants of the towing logic. Panels (c) and (d) illustrate the logic of positive potential, and panels (e) and (f) demonstrate the logic of paste.

Starting with the logic of switching traits, the intuition comes from phenotypic plasticity [14], where the phenotype of an organism changes depending on the environment—for instance, *Candida albicans* [15], a human fungal pathogen exhibits two distinct phenotypic traits depending on the pH. A general situation of switching phenotypical traits can be represented by Fig 2(a) and 2(b), which considers a particular trait of an asexual population represented by a two-dimensional vector that can adopt two different phenotypic forms depending on the environment. Now, if the variable environment is set to be pH for illustration, when the pH is neutral, the potential for the vector corresponding to initial trait configuration could be represented by form (a); when the pH is acidic, the potential shifts to form (b) because the trait adopts a different configuration in acidic environment. In this scenario, even if the state becomes trapped in an undesired local minimum under neutral pH, it can still evolve toward the target position when the environment turns acidic. The estimated time for the algebra to reach the objective position can be calculated using relation (2), with the potential differences derived from form (b).

### 2.2 Second logic: Positive potential

Second, the logic of positive potential, concerns altering the strength, or *weight* of a particular environment potential. This logic corresponds to imposing artificially adjusted environmental pressure to have more rigor and precision compared to the original natural environmental pressure. For instance, although the environmental pressure is applied from the same antibiotics, the shape of the potential differs based on the concentration (which corresponds to weight in this case) of antibiotics [16]. This change can be pictorially represented by form (c) as the initial potential and form (d) as the new potential after applying the logic of positive potential. The time required for the evolutionary reaction can then be estimated using relation (2), with the potential taken from form (c) or (d), based on the DE objective.

### 2.3 Third logic: Paste

Third, the logic of paste concerns expanding the explorable range of the algebra. The intuition comes from DNA elongation in nature through gene duplication [17], which secures additional physical space for new genes to be written. For example, consider an asexual population with only 100 base pairs of DNA, initially positioned at a minimum potential. By adding 100 more base pairs—analogous to “pasting” in sculpture—the population acquires additional genetic material, enabling further evolutionary exploration. In algebraic terms, the logic of paste typically involves increasing the dimension of the algebra. For instance, the initial algebra represented by a one-dimensional variable in Fig 2(e) can be expanded into a two-dimensional space, as shown in Fig 2(f). While no further optimization was possible in the original one-dimensional algebra, the logic of paste enables optimization in the higher-dimensional representation.

### 2.4 Fourth logic: Towing from linkage

Fourth, the logic of towing from linkage relates to the evolution of linked genes, where multiple genes are physically located on the same plasmid or chromosome of a microorganism. This phenomenon is well described by the “hitchhiking effect” [18], in which genes with little selective advantage persist due to their proximity to strongly advantageous genes on the same chromosome. For illustration, consider two genes encoding two two-dimensional vectors that are linked within a plasmid. Even if the first vector tends to move downward along its potential, it may be constrained if the selective advantage of the second vector is much larger, preventing the first vector from moving as expected. In this scenario, even if the first vector becomes trapped in a local minimum, as illustrated in Fig 2(a), mutations corresponding to higher potential can still establish due to the dominant evolutionary influence of the linked second vector. This approach can also stabilize the position of the first vector, effectively *desensitizing* it to potential differences because of the dominant dynamics of the second vector.

### 2.5 Fifth logic: Towing from phenotypic dependence

Fifth, the logic of towing from phenotypic dependence is based on the idea that as one phenotypic trait evolves, it alters the overall fitness landscape and thus the selection pressure on other traits. Mathematically, this situation can be represented by two interdependent algebras, which do not necessarily share genes. In this scenario, the evolution of the first algebra can alter the potential of the second algebra. While generation-independent potentials (i.e., time independent, static potentials) are generally preferred for analytical simplicity when simulating the DE, this logic requires accounting for generation-dependent potentials (i.e., dynamic potentials with respect to time). Thus, to simplify the analysis so that continuously varying potential does not have to be taken into account, one could experimentally restrain the evolution of the second algebra until the first algebra completes its influence on the second algebra’s potential, and then allow the second algebra evolution to proceed. Note that under this approach, the total number of generations for the evolutionary reaction includes the additional duration required for the potential change.

### 2.6 Sixth logic: Towing from pleiotropy

Sixth, the logic of towing from pleiotropy exploits the fact that a single gene can influence multiple phenotypical traits (or algebras) [19]. This logic is structurally similar to the logic of linked genes, where the dominant algebra determines the evolutionary trajectory of a less strongly selected algebra. However, unlike the linked case, the effect in pleiotropy is more direct: the weaker-selected algebra must move whenever the dominant algebra moves. The evolutionary time in this scenario can be estimated by considering the dominant algebra’s evolution via relation (2) and its effect on the DNA sequence, which subsequently drives the evolution of the target algebra. For precise modeling, the relationship between the DNA sequence and the algebraic representation of the corresponding phenotypes must be clearly defined.

Note that the three variants of the logic of towing (fourth, fifth, and sixth) are illustrated in Fig 2(a) and 2(b): Fig 2(a) depicts the primary algebra of interest, while (b) shows the secondary algebra that facilitates towing.

### 2.7 Seventh logic: Negative potential

**Fig 3:**
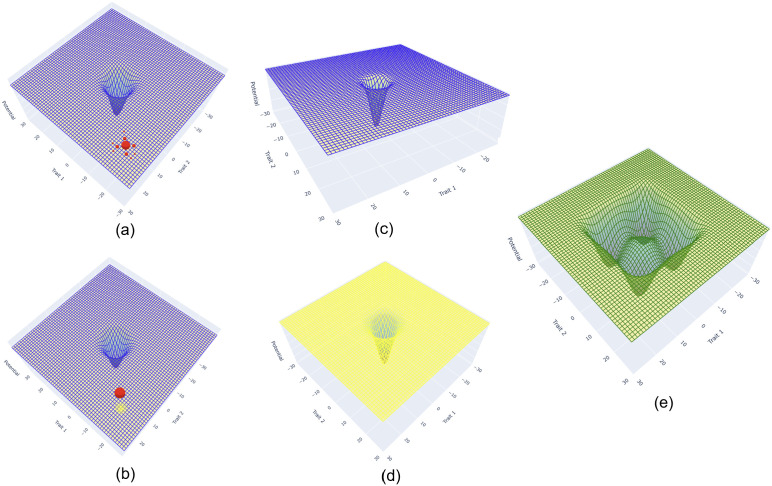
Continuous pictorial representation of the logics in the 2D vector case. Panels (a) and (b) represent the logic of negative potential. Panels (c), (d), and (e) illustrate the logic of emergence.

Seventh, the logic of negative potential is inspired by the phenomenon of evolutionary rescue [20], often observed in antibiotic-resistant bacteria. When a strong antibiotic is applied, most mutations die off except for a few that can withstand the antibiotic [21]. Since there are few competing mutations among the survivors, the establishment probability for these remaining variants increases significantly. Thus, a harsh environment can act as a precise filter to select the desired trait. This harsh environment is represented as a negative potential, illustrated in (a) and (b) of Fig 3. In (a), the radius of the ball represents the proportion of mutations, showing that only a few vectors occupy the position (−16,16). After applying the negative potential, which permits survival only at (−16,16), the original population around (−20,20), shown by the dotted yellow sphere, is driven to (−16,16), illustrated by the solid red sphere. The benefits of this logic are twofold. First, it accelerates evolution by reducing the time required for beneficial mutations to establish and fix compared to the case where there are lot of competing lineages. Second, it enables precise selection so that the establishment of key intermediate states could be secured. In this case, assuming that the time taken for establishment is negligible after survival, the total evolutionary time can be estimated as $t=\log_{2}(N/N_{initial})$, representing the time required for the surviving population to double from $N_{initial}$.

### 2.8 Eighth logic: Emergence

Eighth, the logic of emergence concerns the combination of environmental potentials that produces emergent effects. It is often assumed that when two potentials, $\Phi_{1}$ and $\Phi_{2}$, take the forms shown in Fig 3(c) and 3(d), their sum $\Phi_{sum}$ can be expressed as a linear addition, $\Phi_{sum}=\Phi_{1}+\Phi_{2}$. However, some potentials may exhibit emergent properties, such that $\Phi_{sum}=\Phi_{1}+\Phi_{2}+f(\Phi_{1},\Phi_{2})$, where $f(\Phi_{1},\Phi_{2})$ is an emergent function, resulting in the form illustrated in (e). Applying this logic in directed evolution requires extensive experimental data to accurately construct the emergent potential. The expected evolutionary time can then be estimated using relation (2), from incorporating the information of emergent potential.

### 2.9 Ninth logic: Varying the mutation rate

Ninth, the logic of varying the mutation rate concerns adjusting the mutation rate of the population. Since the mutation rate strongly influences the evolutionary trajectory, strategically increasing or decreasing it at specific stages can guide the population more efficiently along the desired path. Experimentally, varying the mutation rate *in vivo* can be achieved using various methods [22–24].

### 2.10 Tenth logic: Explicit modification

Tenth, the logic of explicit modification involves directly moving the red ball to or near the target (white ball) position. In practice, this can be realized through genetic engineering, effectively placing the population closer to the desired trait. Such an approach can benefit from genomic design within the framework of synthetic biology (e.g., [25]).

To demonstrate how to use the introduced logics by directed evolution (DE) simulation, the traits of interest was modeled as an $n_{1}\times n_{2}\times n_{3}$ discretized matrix *M*, which is essentially an application of algebraic modeling to directed evolution (DE) [8]. This matrix contains only 0s and 1s, where 1s correspond to points defining the physical shape in three-dimensional space. Rather than using DNA sequences as the basis for mutation, mutations are applied directly to the algebra *M* representing the phenotypic trait, such that an element of *M* changes from 0 to 1. Mathematically, the algebra is defined as


$$\begin{array}{c} A=(\{M_{i,j,k}\in \{0,1\}:i\in \{0,\ldots ,n_{1}\},j\in \{0,\ldots ,n_{2}\},k\in \{0,\ldots ,n_{3}\}\},+). \end{array}$$

**Fig 4:**
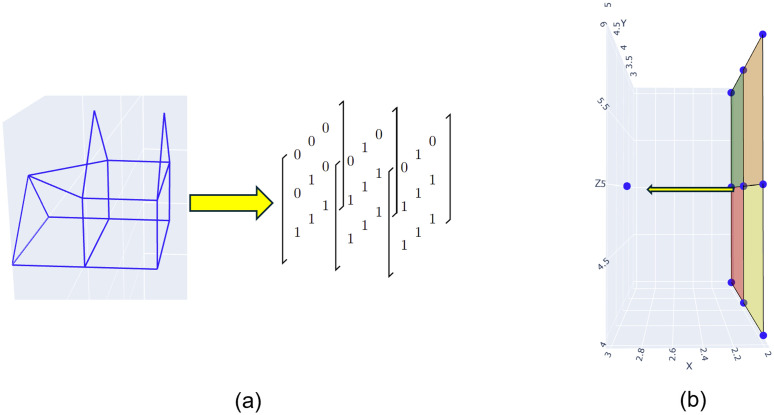
Pictorial illustrations of the introduced concepts. (a) illustrates an example of discretization, while (b) represents the square-based mutation rule.

Although other algebraic representations could be used, this $n_{1}\times n_{2}\times n_{3}$ matrix is chosen to intuitively capture the continuous physiological geometry of the traits in three-dimensional space through discretization, as illustrated in Fig 4.

With the defined algebra, consider a scenario in which the limb of an imaginary asexual population gradually evolves into a wing. To explicitly construct this process of wing evolution, studies on the evolutionary development of wings in birds, insects, and bats are used to define the potentials. Specifically, the development of the patagium enabling thermoregulation [26,27], the semi-wing structure allowing proto-birds to generate thrust for climbing trees [28], and the wing structure enabling lift for flight [29] are incorporated. Note that the initial discretized matrix represents the shape of the limb.

For the matrix, the mutation law is defined as follows: a mutation occurs when an element 0 changes to 1. This mutation occurs only if there is a specific distribution of 1s adjacent to the mutation direction, representing the intuitive fact that new morphological parts form only from existing structures. For example, when the matrix evolves in the *x*-direction at position *M*_3,4,5_, one of the following sets of elements must already be equal to 1:


$$\begin{array}{c} \begin{array}{c} M_{2,4,5}=M_{2,3,5}=M_{2,3,4}=M_{2,4,4},\;M_{2,4,5}=M_{2,3,5}=M_{2,3,6}=M_{2,4,6},\\ M_{2,4,5}=M_{2,4,6}=M_{2,5,6}=M_{2,5,5},\;M_{2,4,5}=M_{2,5,5}=M_{2,5,4}=M_{2,4,4} \end{array} \end{array}$$

as illustrated in Fig 4(b). While other mutation rules could be applied, this choice represents a scenario in which a mutation occurs only when sufficient prior phenotypic material, or “paste,” exists in the matrix.

With a well-defined matrix and mutation law, the evolutionary path depends on the specific information encoded in the potentials. The thermal regulation potential is approximately modeled as


$$\begin{array}{c} \Phi_{thermal}-\Phi_{thermal\;max} \end{array}$$

$$\begin{array}{c} \begin{array}{c} \approx \;\left\{ \begin{array}{cc} -\sum_{i=0}^{N_{i}}\sum_{k=0}^{N_{k}}\sum_{j=0}^{N_{j}}M_{i\;j\;k}\cdot (y_{criteria,\;thermal}-j{)}^{\frac{1}{4}}\cdot w_{consecutive\;y,\;thermal}\cdot s\cdot w_{thermal,\;thermal} & \text{if }M_{i,\;j+1,\;k}=1\;\text{or}\;M_{i,\;j-1,\;k}=1,\\ -\sum_{i=0}^{N_{i}}\sum_{k=0}^{N_{k}}\sum_{j=0}^{N_{j}}M_{i\;j\;k}\cdot (y_{criteria,\;thermal}-j{)}^{\frac{1}{4}}\cdot w_{else,\;thermal}\cdot s\cdot w_{thermal,\;thermal} & \text{otherwise.} \end{array}\right. \end{array} \end{array}$$

The thrust potential is defined as


$$\begin{array}{c} \Phi_{thrust}(M_{ijk})-\Phi_{thrust\;max}\approx \left(-\sum_{j=0}^{N_{j}}({Weighted\;Area}_{x}{)}_{j}\cdot w_{thrust,\;thrust}\right)s, \end{array}$$

where the weighted area is the *x*-plane (i.e.,*YZ*-plane) projected area:


$$\begin{array}{c} {Weighted\;Area}_{x}=\left\{ \begin{array}{cc} 0, & (z_{\max}-z_{\min}{)}_{j}<4,\\ N(x-\mathrm{plane projected points})+\sum_{k}\sum_{j}w_{consecutive\;y,\;thrust}\;M_{i,j,k}M_{i,j+1,k}\\ \;+\sum_{k}\sum_{j}w_{wide\;z,\;thrust}\;M_{i,j,k}\;|k-z_{middle}|, & (z_{\max}-z_{\min}{)}_{j}\geq 4. \end{array}\right. \end{array}$$

Note that the weighted area is set to be nonzero only if $(z_{\max}-z_{\min}{)}_{j}$ is greater than or equal to 4, based on the assumption that thrust can be meaningfully generated only when the wing width is sufficiently large. Moreover, the second term of the weighted area rewards continuously connected points to favor a wing without holes, while the third term rewards structures that extend further away from $z_{middle}$.

The mass is defined as


$$\begin{array}{c} m=N(\text{total elements})\cdot w_{mass\;factor}. \end{array}$$

The lift potential is defined as


$$\begin{array}{c} \Phi_{lift}(M_{ijk})-\Phi_{lift\;max}\approx -\sum_{j=0}^{N_{j}}(\Delta P\cdot {Area}_{x}{)}_{j}\cdot s, \end{array}$$

where


$$\begin{array}{c} (\Delta P{)}_{j}=[\frac{\rho_{air}}{2}(v_{fast}^{2}-v_{slow}^{2}){]}_{j}\approx [\frac{\rho_{air}}{2}(\frac{l_{long}^{2}}{l_{short}^{2}}-1)v^{2}{]}_{j}. \end{array}$$

Here, $(\Delta P{)}_{j}$ corresponds to the pressure difference of the wing derived from Bernoulli lift for each *Y*-slice (i.e., each *j* value), while $({Area}_{x}{)}_{j}=\{(z_{\max}-z_{\min})\times 1{\}}_{j}$ is the area of the wing slice at *y* = *j*, projected onto the plane $x=x_{\min}$ (i.e., the portion of *YZ* plane).

The towing potential is defined as


$$\begin{array}{c} \Phi_{towing}=N(l\geq l_{criteria})\cdot s\cdot w_{towing}, \end{array}$$ (3)

where *l* = *i*, *j*, or *k*, depending on the intended towing direction. With this formulation, the potential decreases (i.e., the fitness increases) for matrices that contain 1s aligned with the direction of interest, particularly when their coordinates along the chosen axis exceed $l_{criteria}$.

The emergent potential, which is not used in this simulation, is


$$\begin{array}{c} \Delta \Phi_{emergence}=w_{thermal,\;emergence}(\Delta \Phi_{thermal}{)}^{2}+w_{mobility,\;emergence}(\Delta \Phi_{mobility}{)}^{2}+w_{both,\;emergence}(\Delta \Phi_{thermal}\;\Delta \Phi_{mobility}), \end{array}$$

with


$$\begin{array}{c} {\vec{w}}_{emergence}=[w_{thermal,\;emergence},\;w_{mobility,\;emergence},\;w_{both,\;emergence}]. \end{array}$$

Within this potential setting, several observations can be made. When only $\Phi_{thermal}$ is active, the optimal configuration of the evolved structure has all elements with $j\leq y_{criteria,\;thermal}$ equal to 1. When only $\Phi_{thrust}$ is applied, multiple optimal configurations exist that maximize the *x*-plane projected area. When only $\Phi_{lift}$ is active, the matrix evolves toward maximizing the ratio $l_{long}/l_{short}$.

These optimal configurations from different potentials differ significantly, highlighting the need to appropriately adjust the potentials to guide the system toward the target matrix shown in Fig 4(b).

The total potential is defined as


$$\begin{array}{c} \Phi_{total}=w_{mobility,\;total}\cdot \Phi_{mobility}+w_{thermal,\;total}\cdot \Phi_{thermal}+\Phi_{towing}+\Phi_{emergence}, \end{array}$$

where


$$\begin{array}{c} \Phi_{mobility}=w_{thrust,\;mobility}\cdot \Phi_{thrust}+w_{lift,\;mobility}\cdot \Phi_{lift}. \end{array}$$

Note that some technical details for defining the potentials (e.g., constraints preventing overly triangular shapes or excessively thick wings) are omitted here, as too much detail may obscure the main message. Readers interested in these details could refer to the codes provided.

**Fig 5:**
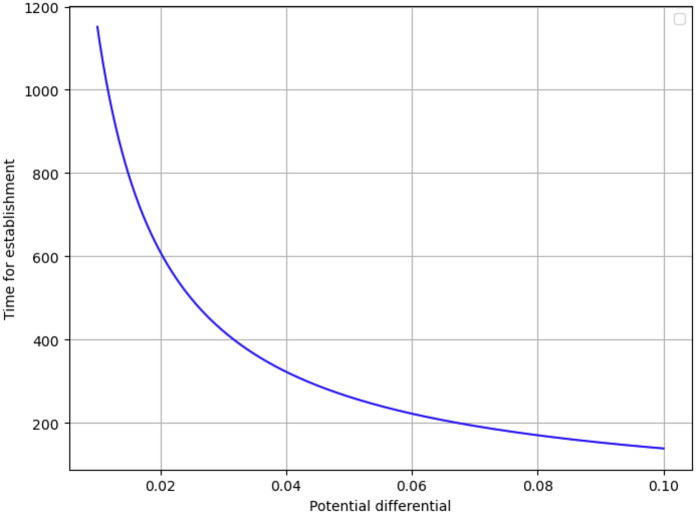
Relation between ΔΦ and establishment time. The assumptions are $\Delta \Phi_{A_{i}\leftarrow A_{i-1}}\approx \Delta \Phi_{A_{i+1}\leftarrow A_{i}}$ and $m_{A_{i}\leftarrow A_{i-1}}=10^{-7}$ for i∈{1,…,T}. The graph depicts the time required (y-axis) for the establishment of a beneficial mutation that reduces the potential to a specified magnitude (x-axis).

Table 1 shows the initial weight values that determine the total potential. These weights are experimentally adjustable parameters, tuned from applying diverse quantitative logics introduced above. They are set such that each mutation approximately lowers the potential by ΔΦ≈−0.01~−0.1, consistent with empirical observations of natural evolution [30,31]. Under these conditions, each newly established mutation requires approximately T≈300 generations (just roughly assuming that the new mutation lowers the potential by ~−0.05) according to equation (2). This result is based on an assumed mutation rate of $m\sim 10^{-7}$, which lies between previously reported values of $m\sim 10^{-9}$ [30] and $m\sim 10^{-5}$ [32], and thus represents an experimentally attainable value, while k≈1 [8]. A pictorial representation of the relationship described by equation (2), under the assumptions that $m\approx 10^{-7}$, $\Delta \Phi_{A_{i}\leftarrow A_{i-1}}\approx \Delta \Phi_{A_{i+1}\leftarrow A_{i}}$, and $m_{A_{i}\leftarrow A_{i-1}}=10^{-7}$ for i∈1,…,T, is shown in Fig 5.

**Table 1:** Initial weights. The weights are set such that mutations decrease the potential on the order of ΔΦ≈−0.01~−0.1.

Weight	Value
$w_{mass\;factor}$	1/50
*g*	10
$w_{thrust,\;mobility}$	1/5
$w_{lift,\;mobility}$	1/40
$w_{thermal,\;total}$	1/3
$w_{mobility,\;total}$	2/3
$y_{criteria,\;thermal}$	10
$w_{thermal,\;thermal}$	1/2
$w_{consecutive\;y,\;thermal}$	10
$w_{else,\;thermal}$	5
$w_{thrust,\;thrust}$	12.5
$w_{consecutive\;y,\;thrust}$	1/5
$w_{wide\;z,\;thrust}$	1/10
$w_{velocity,\;lift}$	1
$w_{towing,\;x}$	0
$w_{towing,\;y}$	0
$w_{towing,\;z}$	0
$w_{criteria,\;towing,\;x}$	100
$w_{criteria,\;towing,\;y}$	100
$w_{criteria,\;towing,\;z}$	100
${\vec{w}}_{emergence}$	$\vec{0}$

**Fig 6:**
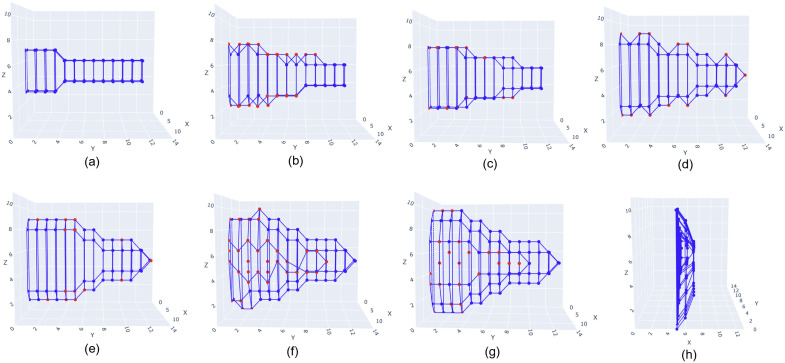
Graphical representation of approximated milestones in the directed evolution of the discretized matrix. The red dots indicate elements that evolved from the preceding objective. The *x*-, *y*-, and *z*-axes represent the spatial axes corresponding to the physical morphology of the wing.

Now, the initial limb shown in Fig 6(a) contains 94 elements equal to 1, while the final wing in Fig 6(g) contains 214 such elements. A rough estimate therefore indicates that the transition from limb to wing requires approximately 214−94=120 mutational establishments, corresponding to about $120\times 300\sim 3.6\times 10^{4}$ generations. Even assuming a generation time of roughly 30 minutes for an asexual population, the entire process would require approximately 25 months, making it economically impractical. Furthermore, the evolutionary trajectory is not guaranteed to reach the target matrix, since many alternative mutational paths exist and evolutionary outcomes are irreversible.

To overcome this inefficiency, the logics introduced above can be employed to design the evolutionary pathway. In doing so, several fundamental principles should be considered. First, the matrix is more likely to evolve in the direction that maximally decreases the potential, i.e., along the −∇Φ direction, as implied by equation (1). Second, to minimize the reaction time, the evolutionary path should ideally follow a trajectory of approximately uniform potential decrease [8], unless the logics provide effective shortcuts. Based on these principles, the evolutionary pathway guided by the logics is designed to achieve potential decreases around ΔΦ≈−0.1~−0.4 (but often going beyond this range of potential decrease). Such a level of potential decrease may be experimentally feasible given the relatively strong selection pressures observed in bacteria [33].

In summary, during the DE process, the weights assigned to the potentials are systematically adjusted to satisfy two conditions: First, if a mutation introduces a new point, the probability of establishment increases when that point lies within the desired region. Second, the reduction in potential associated with the added point falls within the target range of ΔΦ≈−0.1~−0.4, thereby contributing effectively to the overall decrease in potential.

Moreover, a realistic beneficial mutation rate of *m* = 10^−7^ is adopted for the same reasons discussed above. Under these parameters, the estimated evolutionary time decreases to approximately 3,000−16,500 generations, corresponding to roughly 3–11.5 months. This theoretically demonstrates that the establishment time for new mutations reduces when the logics are used.

Note that the population evolution in this setting appears to deviate from the power-law behavior [31] of selection coefficient accumulation. Despite this seemingly apparent discrepancy, the evolution still fundamentally follows a power law; the observed linear behavior is possible because it corresponds to the initial regime of the power-law curve. Over a sufficiently large number of generations, the matrix evolution is expected to converge to the expected power-law trend.

The approximated objective evolutionary path is illustrated in Fig 6. Note that this path is only approximate, as each subgoal from (b) to (g) represents an ideal target that is unlikely to be achieved exactly because of the stochastic nature of evolution. The evolutionary process begins by adjusting the weights according to S1 Table in S1 Appendix so that thrust force acts as the dominant environmental pressure for 3,000 generations, corresponding to stage (a)→(b). The weights are then modified according to S2 Table in S1 Appendix so that thermal regulation becomes the primary selective factor for 2,000 generations, corresponding to stage (b)→(c). Subsequently, the thrust- and thermal-dominated selective pressures are repeated for one additional cycle: stage (c)→(d), which follows the weights in S3 Table in S1 Appendix (using the same weights as in stage (a)→(b)) lasts for 2,500 generations, whereas stage (d)→(e) which follows the weights in S4 Table in S1 Appendix (using weights similar to those of stage (b)→(c)) lasts for 1,500 generations. Finally, the weights corresponding to increasing selection for lift force, given in S5 Table in S1 Appendix, are applied during stages (e)→(f)→(g) for 4,000 generations. To realize this qualitative design, the logic of towing and the logic of positive potential are actively employed. Specifically, by using these logics, weights are adjusted to approximately maximize the potential drop along the desired mutation direction, thereby increasing the probability of establishment in that direction. The tables listing the specific weights used for each stage are provided in the Appendix.

Moreover, the logic of negative potential is applied at transitions between objectives (i.e., at generations 3,000, 5,000, 7,500, and 9,000). In this approach, evolution toward the new objective (e.g.,(b)→(c)) starts from only one of the resulting traits from the previous objective (e.g., (a)→(b)). Although this step is not strictly necessary, it is introduced to improve the reliability of the simulation computational process. In rare cases, due to unexplained issues, weights updated by the logics in were not properly reflected when attempting to perform the full 13,000-generation DE simulation in a single run. To ensure computational reliability, the simulation is therefore stopped after each objective stage and restarted using the result from the previous stage. In this process, a single trait from the previous stage is selected as the initial matrix for the next stage, which is effectively equivalent to imposing a negative potential.

The matrix corresponding to Fig 6(g) is selected as the final reference objective among several plausible divergent structures because it preserves the key morphological features defining a wing. Moreover, the morphology in (g) aligns with a rough quantitative expectation: if the weights are set such that typical mutations produce a potential change of approximately ΔΦ≈−0.15, then relation (2) predicts that the final structure incorporates about 120 new points over roughly 13,000 generations. Note that the preliminary iterations were performed to predict the final wing shape under weight sets that typically produce mutations with |ΔΦ|~0.1 to 0.4 (but occasionally existing beyond this range) and direct the evolution toward a morphology resembling Fig 6(g). The outcomes of these preliminary simulations were then used to estimate the expected final wing structure, supporting the selection of Fig 6(g) as the representative form. Therefore, the matrix in Fig 6(g) serves as an informed reference that both reflects the desired wing shape and is consistent with rough theoretical predictions.

As a side note, in Fig 6, the edges are drawn to approximate the overall morphology so that its shape can be perceived intuitively, rather than to represent it rigorously. In other words, given the set of dots (i.e., the 1s in the discretized matrix) that constitute the morphology, there are multiple valid ways to connect them to form edges. The edges shown here—connecting the boundary vertices—represent just one such choice. Therefore, for a rigorous morphology-relevant analysis, one should disregard these edges and consider only the vertices within the wing structure. For instance, quantities such as the lift potential may be inaccurately estimated if geometric measures—such as the length of an x-plane cross section—are derived from the depicted edges; instead, such measures should be computed directly from the vertices (i.e., ignoring the edge representation).

However, since this level of geometric precision was not the focus of this article, approximate edges were retained to enhance readability. For readers particularly interested in the exact morphology—where a complete representation of all dots is essential—the provided code can be used to visualize the full set of points within the bulk (shown as purple dots), as also illustrated in Fig 8.

**Fig 7:**
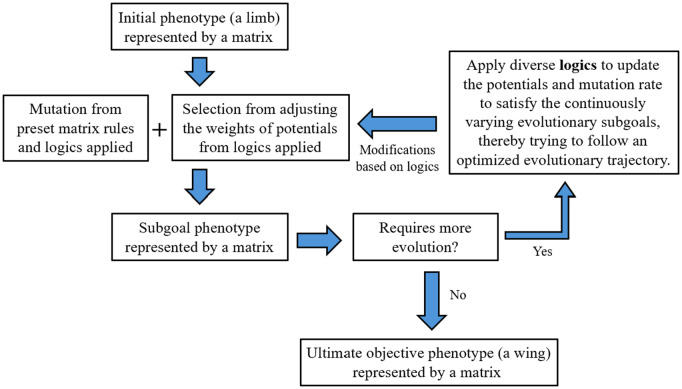
Flowchart for the DE process using quantitative logics. The quantitative logics are applied to steer the evolutionary trajectory toward the target trait. The flowchart focuses on the specific example of wing evolution from a limb represented by a discretized matrix.

In summary, the flowchart illustrating how the quantitative logics can be utilized in the DE process—particularly for the discretized matrix representation of wing evolution—is presented in Fig 7. In the simulation, all towing-related logics are incorporated into a single “logic of towing,” while the logic of switching traits is included within the logic of positive potential. The logics of explicit modification, varying mutation rate, paste, and emergence are not applied in this instance, as they are unnecessary for evolving the wing; however, they remain available and can be simulated using the provided code.

The simulation is developed based on the operator model [13], which enables intuitive calculation of stochastic evolutionary processes. Within this framework, mutation and drift—the components involving randomness—are naturally incorporated without requiring additional implementation effort.

## 3 Results

**Fig 8:**
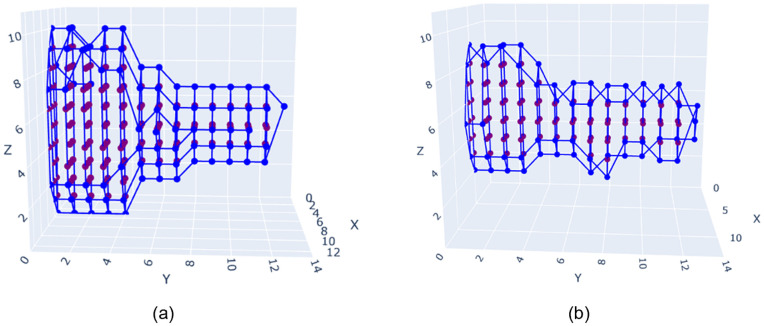
Resulting wing morphology examples for experimental and control group. The result (a) is for the experimental group which used logics during DE process while the result (b) is for the control group which did not utilize any logic.

Fig 8 represents an example of the final result for each of the experimental and control groups after 13,000 generations of directed evolution. The experimental group example at Fig 8(a) is chosen that had highest success rate among 30 independent simulation results while the control group example at Fig 8(b) was chosen randomly among the 30 independent simulation results. Note that in this figure, whole dots within the resulting morphology were expressed (purple dots for representing the dots that do not directly involve for defining edges) to give a sense for how the evolution worked out.

The simulation results for a 13,000-generation evolution of experimental group (i.e., group with logics applied during DE process) approximately follow the pre-designed trajectory, with the target wing structure shown in Fig 6(g) (the resulting wing morphology from experimental group that utilized logic is provided in the Appendix). Table 2 summarizes the results from 30 independent simulations applying the logics for 13,000 generations and 30 independent simulations using only the initial evolutionary parameters without applying logics. Note that from “ΔTotal Number of 1s,” and the previous fact that the objective wing structure has total 214 “1s,” approximately 129 new mutations on average are established for the experimental group. From such information, the establishment time per “0→1” could be found to be about ~101 generations. As a side note, a total of 31 simulation results were made for the experimental group, but only randomly chosen 30 results were used to maintain a consistent sample size. The omitted result (the final simulation) was arbitrarily chosen for exclusion and not based on its content or characteristics. For the control group, 3 out of 33 simulation results are excluded using the interquartile range (IQR) method, as they exhibit outlier behavior in ΔLift Force (values beyond the 3× IQR range). The average number of new 1s fixed in the control case over 13,000 generations is approximately 50, corresponding to occurrence of one mutation fixation for every ~260 generations. Referring to equation (2) and Fig 5, this implies a potential decrease of approximately 0.05 per added 1, consistent with predictions based on the initial weights in Table 1.

**Table 2:** Quantities measured from 30 independent simulations using the logics (experimental group) and 30 independent simulations without applying logics (control group). “Success Rate” refers to the percentage of 1s overlapping with the objective reference trait that appear in the resulting matrix, whereas “ΔTotal Number of 1s” denotes the percentage difference between the total number of 1s (without the consideration for overlapping) in the resulting matrix and that in the objective reference matrix. Differences in lift force, thrust force, and thermal regulation between the resulting matrix and the objective reference matrix are also reported. The percentage difference is computed as $\frac{Resulting\;matrix\;quantity-Objective\;matrix\;quantity}{Objective\;matrix\;quantity}\times 100(\%)$.

Comparing Quantity	Experimental Average	Experimental Stdev	Control Average	Control Stdev
Success Rate	82%	3.6%	63%	1.6%
ΔTotal Number of 1s	0.1%	2.6%	−33%	0.99%
ΔLift Force	3.4%	16%	−91%	6.4%
ΔThrust Force	2.7%	7.1%	−18%	2.5%
ΔThermal Regulation	4.1%	8.7%	−43%	7.2%

When calculating the Δ values for lift, thrust, and thermal regulation, the weights listed in S5 Table in S1 Appendix—corresponding to the environmental conditions at the final stage (i.e., (f)→(g) step in Fig 6)—are used. For example, ΔLift Force is calculated as $\frac{\Phi_{lift}(M_{resulting})-\Phi_{lift}(M_{objective})}{\Phi_{lift}(M_{objective})}$, where $\Phi_{lift}(M)$ is evaluated using the weights from S5 Table in S1 Appendix.

It is also observed that, the standard deviations of the experimental group for each quantity in Table 2 are larger than those of the control group. Additionally, the standard deviations associated with $\Delta \Phi_{Lift},\;\Phi_{Thrust},\;\Phi_{Thermal}$-related quantities are larger than those for the success rate and ΔTotal Number of 1s in both experimental and control groups.

Table 3 shows that the experimental group (with logics) and the control group (without logics) are clearly distinguishable. The p-values for comparing mean values for each group, obtained using Welch’s t-test, are extremely close to zero, indicating strong statistical separation between the groups. Furthermore, Cohen’s *d* values for all quantities are significantly greater than 2, demonstrating that the two distributions are almost entirely non-overlapping.

**Table 3:** Statistical analysis comparing the experimental and control groups. The comparison is based on simulation results with logics (30 results) and without logics (30 results). Welch’s t-test is used to compute p-values, and Cohen’s *d* is used to quantify effect size.

Comparing Quantity	p-value	Cohen’s d
Success Rate	$9.5\times 10^{-28}$	6.5
ΔTotal Number of 1s	$9.5\times 10^{-40}$	15
ΔLift Force	$4.3\times 10^{-28}$	7.1
ΔThrust Force	$5.8\times 10^{-17}$	3.5
ΔThermal Regulation	$2.4\times 10^{-30}$	5.8

## 4 Discussion

The results in Table 2 demonstrate that an informed approximation of the objective is feasible, and that the matrix can evolve into traits resembling the target characteristics. In particular, the resulting wings recover, on average, up to 82% of the objective points, while the total number of 1s differs by 0.1% from the expected value. From this result, it can be inferred that evolving the matrix for 13,000 generations is almost the right amount of time required to reach the reference objective. This indicates that, despite the right amount of generations, there remains room for improving the success rate through more refined weight adjustments from using the logics. Moreover, it could be observed that the establishment time for the experimental group (~101) is shortened about 2.6 times compared to the contrast group (~260).

Table 2 further suggests that, for the system to approach the intended morphology, the evolutionary pressures associated with lift, thrust, and thermal regulation should gradually decrease. Moreover, the observed standard deviations indicate that a wider range of final outcomes after 13,000 generation of DE emerges when logics are applied compared to when they are not. Additionally, the larger standard deviations in quantities related to ΔΦ compared to the success rate or ΔTotal Number of 1s imply that it is more difficult to direct evolution to precisely match individual potentials than to achieve the overall number of 1s and the crude morphology of the matrix.

The weight adjustments imposed by the logics were crucial for reaching each subgoal and ultimately the final goal. Specifically, when an experimenter identifies certain degrees of freedom that are permissible for a desired trait (e.g., various wing morphologies that result in the same target lift force in the DE process), adjusting the weights helps constrain this freedom and identify optimal solutions among diverse candidates sharing the same trait. In the present work, the selection of appropriate weights was performed through preliminary manual iterations and anticipation of the final form, which may appear somewhat ad hoc. Therefore, during the process, some mutations were allowed to produce potential decreases outside the *often experimentally observed*, objective range of −0.4≤ΔΦ≤−0.1. Nevertheless, because the present study focuses primarily on the theoretical aspects of the DE process, the potential difference was not required to strictly remain within −0.4≤ΔΦ≤−0.1. Therefore, the preliminary manual tuning procedure was considered sufficient to determine the weights. However, future research would definitely benefit from more advanced methodologies capable of systematically verifying whether the chosen weights ensure that every mutation produces potential changes within the desired range. Such approaches could include automated algorithms that exhaustively evaluate possible evolutionary trajectories through brute-force searches, or AI-driven frameworks that dynamically adjust weights according to the current state of the system based on accumulated environmental and evolutionary data.

Despite the similarity between the simulation outcome and the final objective shown in Fig 6(g), the results in Table 2 do not perfectly match the desired objective for two main reasons: the limited number of weight adjustments during the DE simulation (i.e., only five chances of adjustments as represented by Fig 6), and the restricted number of weights available when optimizing the potentials. If the simulation were extended to evaluate potential values for all plausible mutations at every generation—while simultaneously optimizing the associated weights—the accuracy in achieving the target trait would improve substantially. Such an enhanced system, capable of large-scale computation, would enable the design of more sophisticated evolutionary pathways.

Furthermore, controlling only the three potentials introduced here imposes limitations on achieving the exact target matrix. Multiple divergent evolutionary pathways exist, and in some cases, undesired traits may yield comparable or even greater potential decreases than the objective trait under the preset weights. For example, the symmetry observed in Fig 6(g) cannot be easily enforced, as the defined potentials do not regulate the additional degrees of freedom required to produce symmetric wing geometry. Similarly, evolution along the *x*- and *z*-axes is difficult to control with the limited number of parameters. This is because maintaining ΔΦ within the range −0.1 to −0.4 in the desired direction requires selecting among the most effective available weight combinations (as listed in the Appendix), yet these still permit unintended directional deviations due to insufficient constraints. In summary, more frequent weight adjustments during the DE process and additional potentials providing greater degrees of freedom would be greatly helping to achieve higher-precision DE outcomes. Nevertheless, the primary goal of this work is to demonstrate the feasibility of DE guided by quantitative logics. Accordingly, the implemented code is developed only to the extent necessary for this proof of concept.

Even when |ΔΦ| is relatively large (e.g., 0.3~0.4), a minimum amount of time is still required for mutations to become established. Consequently, if the objective trait requires many such establishment steps, a significant minimum time is unavoidable. To reduce this time, one approach is to begin with naturally existing (or artificially synthesized) traits that are already close to the desired objective, which is to apply the logic of explicit modification. If such a method be practiced, providing a rough structural framework for the desired trait may often be sufficient, as the evolution could be used to refine this rough trait to precisely meet the final objective trait. Conversely, when the behavior of synthesized genes is difficult to predict due to emergent biological effects [34,35], DE methodologies can be used to refine and stabilize these genes. Thus, genetic engineering and directed evolution can function synergistically.

As demonstrated, evolutionary modeling based on discretized trait geometry offers significant advantages for DE calculations. To apply such a matrix framework in practical experiments, it is essential to establish a mutation law that connects DNA-level mutations to changes in the matrix. Ideally, this could be implemented in continuous *in vivo* mutation systems [36–39], where matrix evolution follows predictable rules. A promising direction for future research for such realisation is the development of a *parallelly evolvable genetic system*, consisting of DNA or RNA sequences and regulatory pathways that satisfy four key properties: orthogonality and modularity [40,41], inducibility [42–44], and malleability (evolvability) [45,46]. Orthogonality ensures that multiple genetic information contribute independently to the morphology of the phenotype. From this, the burden for genetic information dependence (e.g., the fingers evolving from the palm structure) could be relieved (e.g., for extreme illustration, the fingers and palm could evolve separately and later be converged in such orthogonal case). Modularity helps prevent unwanted epistasis among gene elements. Inducibility allows specific genetic elements to activate only under defined conditions, supporting controlled matrix evolution. Finally, malleability ensures that genetic elements can adapt under evolutionary pressure. In essence, such a system resembles multiple independent layers of malleable “paste” that are progressively sculpted through evolution, while the logics imposed constrains and guides this process. With such an idealistic genome engineered that satisfy these properties, it becomes possible to envision a *standardized directed evolution* (SDE) system based on discretized matrix representation, in which phenotypic evolution is quantitatively predictable. Further developing such a framework of SDE system would benefit from insight from genotype-phenotype mapping [47–49]. By reducing reliance on case-specific analyses, SDE has the potential to provide a systematic and generalizable foundation for future directed evolution methodologies.

Note that the example above treats the traits defining the algebra as the spatial physiological structure along the *x*-, *y*-, and *z*-directions. Such a setting enables efficient calculation of physical potentials, since both classical mechanics and the trait space can be naturally formulated in three-dimensional space. However, when considering more general traits in arbitrary (often abstract) mathematical spaces, it becomes necessary to develop both theoretical and empirical methodologies to well define a map that relates the abstract trait to the potential.

Moreover, for high-dimensional trait cases (i.e., involving high-dimensional potentials), the optimization process for the DE trajectory becomes computationally more demanding as the dimension *D* increases. This is because the number of matrix components to optimize scales as $N^{D}$, where *N* is the dimension along each axis for the matrix. To address this challenge, rather than continuously optimizing the weights at every step of the DE process, as done in this article, one may reduce the number of optimization steps (i.e., reduce the number of subgoals) at the cost of some acceptable loss in precision. Furthermore, with support from predictive pruning via artificial intelligence-based algorithms trained on extensive DE simulation data, the effectiveness of these fewer optimizations may be enhanced. Additionally, in the future, it could be expected that the optimization process in such high-dimensional potential landscapes, using techniques such as quantum annealing [50,51] or QAOA [52], may be efficiently conducted on future fault-tolerant quantum computing systems [53]. This is because the distribution of population states across different potentials may allow the exploitation of quantum superposition for efficient optimization.

It should be noted that the example of directing wing evolution using a discretized matrix is only one illustration of how quantitative logics can be applied to control the DE process. The logics introduced here can, in principle, be applied to any biological trait evolution, provided that the mutation rules and algebraic representation of the trait are well defined. For such general DE scenarios, including those involving discretized matrices, a rigorous understanding of the genotype–phenotype (GP) map [47–49] is essential for determining both the mutation rules and the algebraic structure of the phenotype. This bottom-up perspective grounds the algebraic properties of the phenotype in underlying gene interactions, enabling the effective application of quantitative logics. As understanding of the GP map improves, the model can be further generalized.

Additionally, since the design of the evolutionary path is highly sensitive to small changes in the potentials, it is crucial to formulate potentials that accurately reflect realistic environmental effects. In reality, because potentials evolve along with the trait, they must be remeasured periodically throughout the DE process. Achieving such required precision in potential estimation will need large datasets from both theoretical and experimental sources, particularly with a detailed understanding of how environmental factors influence the potential.

Lastly, although the wing evolution example presented here is not intended to directly explain real biological wing evolution, it serves as a proof-of-concept demonstration of DE guided by quantitative logics. At a minimum, it suggests that new morphologies can emerge under realistic physical constraints. However, by incorporating more realistic data for mutation rules based on the GP landscape, environmental pressures, and genetic drift, the framework presented here may be extended to explain the realistic evolution of morphological structures (e.g., wings, limbs, or fins). However, it should be noted that the operator model used in this study is designed for asexual populations; extending it to sexual populations would require the inclusion of a sexual reproduction operator $\hat{S}$ [13].

## 5 Conclusion

Through quantitative modeling of logics corresponding to diverse experimental methodologies for addressing challenges in directed evolution (DE) process, it becomes possible to design evolutionary trajectories more effectively. Using a discretized matrix representation of limb geometry, together with well-defined mutation rules and physical potentials, the results demonstrate that these logics significantly improve the efficiency of the DE process. Specifically, the speed of evolution process increases by nearly 2.6 times, while the accuracy of the final trait relative to the objective reaches approximately 82% on average.

These findings suggest that further development of quantitative logics for DE, along with more rigorous formulations of algebraic model corresponding to a trait of interest, could provide deeper insights into how DE should be conducted. Nevertheless, future research is required to develop theoretical and experimental methods for how to construct algebra (such as discretized matrix in this work) that well represent the realistic phenotypical trait, as well as how to automate the DE process. Such advancements are expected to benefit from emerging technologies, including artificial intelligence and quantum computing across a wide range of applications.

## Supporting information

S1 Appendix S1 to S5 tables. (ZIP)
