# Supplementary figures and images for: Quantitative logics for directed evolution of an asexual population
Source: PLoS One. 2026 Jul 29;21(7):e0354488. doi: 10.1371/journal.pone.0354488 (PMC13419203; doi:10.1371/journal.pone.0354488)

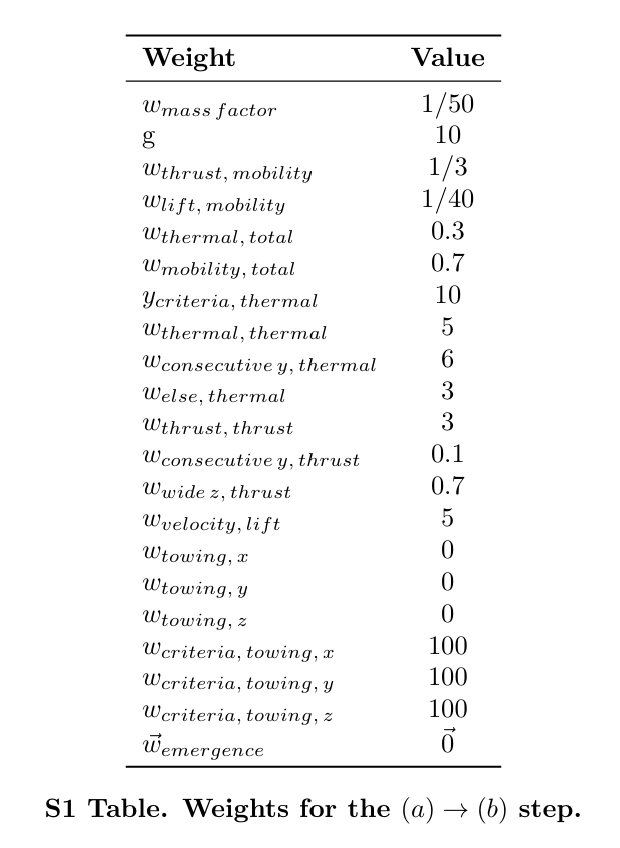

Supplement: S1 Appendix — (ZIP) [file pone.0354488.s001.zip › S1 Appendix/S1 Table.png]

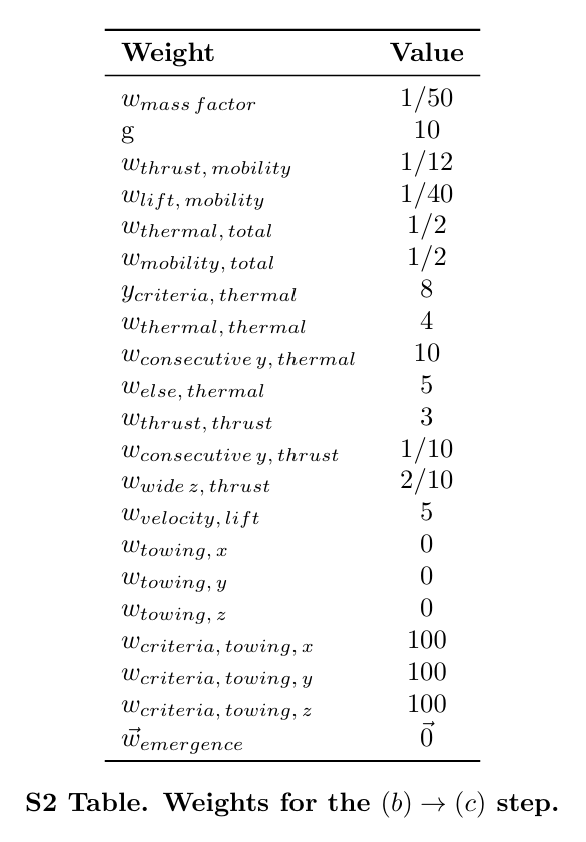

Supplement: S1 Appendix — (ZIP) [file pone.0354488.s001.zip › S1 Appendix/S2 Table.png]

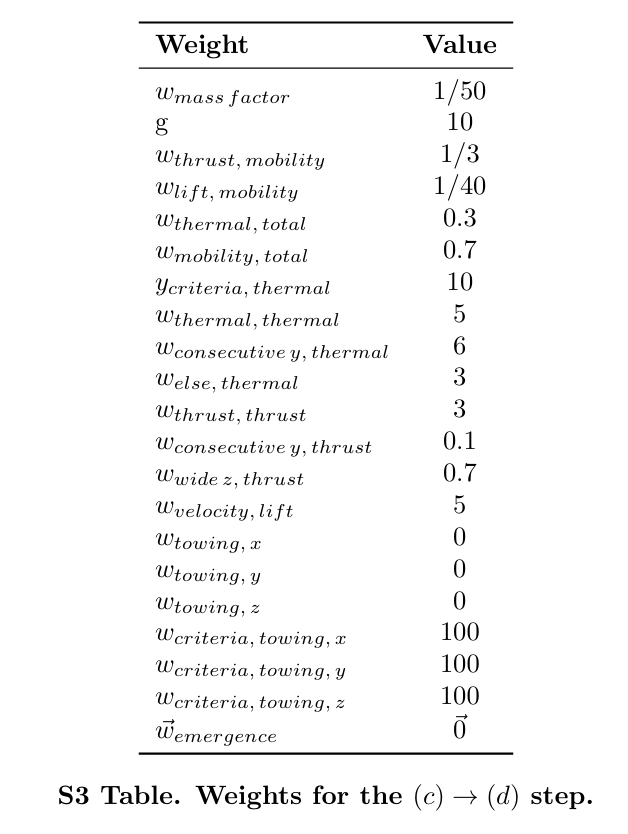

Supplement: S1 Appendix — (ZIP) [file pone.0354488.s001.zip › S1 Appendix/S3 Table.png]

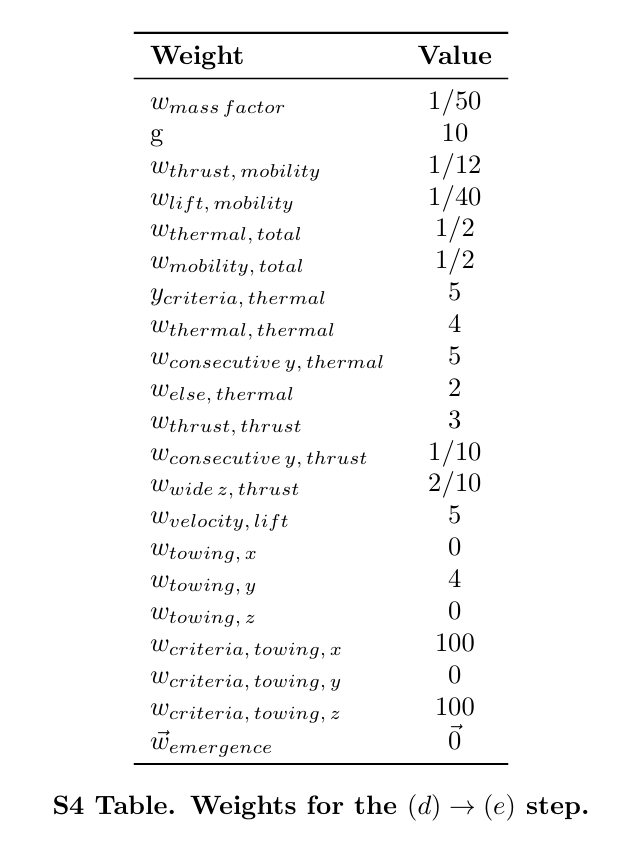

Supplement: S1 Appendix — (ZIP) [file pone.0354488.s001.zip › S1 Appendix/S4 Table.png]

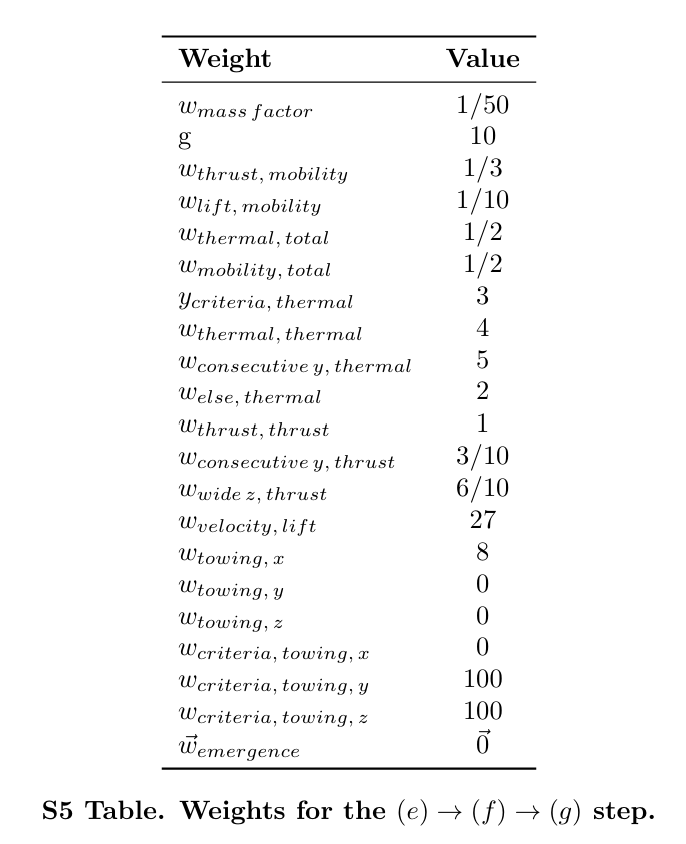

Supplement: S1 Appendix — (ZIP) [file pone.0354488.s001.zip › S1 Appendix/S5 Table.png]
